# Complete plastome sequence of *Tetrataenium Candicans* (tribe Tordylieae, Apiaceae): a medicinal plant

**DOI:** 10.1080/23802359.2019.1674718

**Published:** 2019-10-07

**Authors:** Qun-Ying Xiao, Tu Feng, Yan Yu, Qiang Luo, Xing-Jin He

**Affiliations:** a School of Ecological Engineering, Key Laboratory of Biological Resources and Ecological Remediation of Guizhou Province, Collaborative Innovation Center of Wetland Eco-engineering of Guizhou Province, Guizhou University of Engineering Science, Bijie, P. R. China;; b Key Laboratory of Bio-Resources and EcoEnvironment of Ministry of Education, College of Life Sciences, Sichuan University, Chengdu, P. R. China

**Keywords:** *Tetrataenium candicans*, complete chloroplast genome, phylogenetic analysis

## Abstract

*Tetrataenium candicans* is a Himalayan native medicinal plant species. In this study, we report and characterize the complete plastid genome sequence of *T. candicans* in order to provide genomic resources helpful for promoting its systematics research and conservation. The complete chloroplast (cp) genome is a circular structure and 147,335 bp in length, composing of one large single-copy (LSC) region of 92,996 bp, one small single-copy (SSC) region of 17,473 bp, and separated by a pair of inverted repeat (IR) regions of 18,433 bp each. It encodes 129 genes, including 85 protein-coding genes, 36 tRNA genes, and 8 rRNA genes. The GC content is 37.5%. Phylogenetic analysis of 31 representative plastomes indicated that the *T. candicans* was close to *Semenovia gyirongensis*.

*Tetrataenium candicans* (Wallich ex de Candolle) Mandenova is a widespread species of the Himalayan in the family Apiaceae. It grows in the sparse forests, coniferous forests margin, scrub on arid slopes and in abandoned fields, streamsides with altitudes ranging from 1800 to 4500 m (Pu and Watson 2005). It is a native medicinal herb being a potential source of Xanthotoxin and has constant demand in pharmaceutical industries (BCIL 1996). Over-collecting coupled with other biotic pressures posed a severe threat to its existence in natural habitats and thus, enlisted as Endangered species of the Himalayan region (CAMP 2003).

The species was first described by Candolle (1830) as *Heracleum candicans* Wall. ex DC. within *Heracleum* L. section *Tetrataenium*. Later, *Tetrataenium* was elevated to generic rank (Mandenova 1959) and it was also transferred to *Tetrataenium* as *T. candicans* (Wallich ex de Candolle) Mandenova. Paik (2008) recognized that *T. candicans* may represent a new genus. Recent researches proved that *Tetrataenium* was not monophyletic including *Tetrataenium* sensu stricto and *Candicans* clade (Downie et al. 2010; Logacheva et al. 2010; Yu et al. 2011; Liu and Downie 2017). Consequently, the genetic and genomic information is urgently needed to promote its systematic research and the development of the conservation value of *T. candicans.* Here, we determined to report the complete plastid genome sequence of *T. candicans* (GenBank accession number: MN419228) to provide genetic and genomic information to promote its systematics research and conservation.

The mature leaves of *T. candicans* were obtained from a scrub on arid slope near Tiantuo town (29°53′33.52′′N, 97°38′13.05′′E, altitude 4074 m), Zuogong County, Xizang, China. Voucher specimens (voucher number: xqy201408260101) were deposited in the herbarium of Natural History Museum of Sichuan University (SZ). The experiment procedure is as reported in Xiao et al. (2019). Around 5 Gb raw data were assembled against the plastome of *Semenovia gyirongensis* (MK757488) (Xiao et al. 2019) using Geneious version 11.0.4 (Kearse et al. 2012). The plastome was annotated using Geneious version 11.0.4 against the plastome of *S. gyirongensis* (MK757488) coupled with manual check and adjustment.

The plastome of *T. candicans* was found to possess a total length 147,335 bp with the typical quadripartite structure of angiosperms, containing a large single-copy region (LSC) of 92,996bp and a small single-copy region (SSC) of 17,473 bp jointed by two identical inverted repeat regions (IRa and IRb, 18,433 bp each). A total of 129 genes are successfully annotated, consisting of 85 protein-coding genes, 8 rRNA genes, and 36 tRNA genes were annotated. Among them, 15 gene duplicates in the inverted repeat (IR) regions include six tRNA, four rRNA, and five protein-coding genes. Total GC content is 37.5%.

We used RAxML (Stamatakis 2014) with 1000 bootstraps under the GTRGAMMAI substitution model to reconstruct a maximum likelihood (ML) phylogeny of 31 published complete plastomes of Apioideae, using *Bupleurum latissimum, B*. *boissieuanum*, and *B*. *falcatum* as outgroups. The phylogenetic tree (Figure 1) indicated that *T. candicans* was closely related to *S. gyirongensis*. This published *T. candicans* chloroplast genome will provide useful information for phylogenetic studies and conservation genetics.

**Figure 1.**
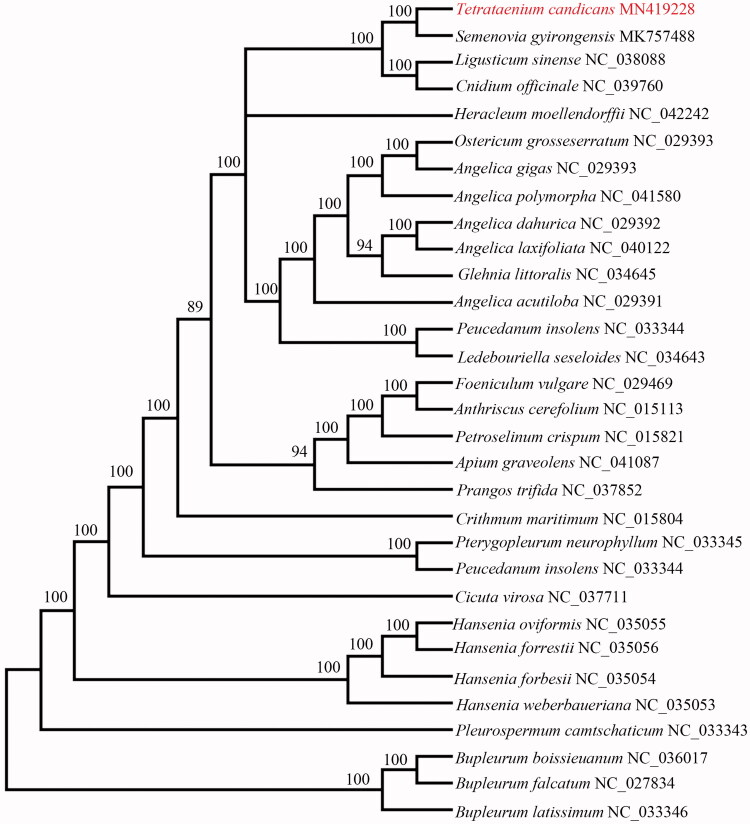
The best ML phylogeny recovered from 31 complete plastome sequences by RAxML. The number on each node indicates bootstrap support value.

## Ethical approval

Research involving human participants and/or animals, this article does not contain any studies with human participants or animals performed by any of the authors.

## References

[CIT0001] BCIL. 1996 Sectoral study of Indian medicinal plants- status, perspective and strategy for growth. New Delhi, India: Biotech Consortium India Ltd.

[CIT0002] CAMP. 2003 CAMP report: conservation assessment and management prioritisation for the medicinal plants of Jammu and Kashmir, Himachal Pradesh and Uttaranchal, Workshop, Shimla, Himachal Pradesh. Vol. 206 Bangalore, India: FRLHT.

[CIT0003] Candolle D 1830 Umbelliferae In: De Candolle AP, editor. Prodromus systematis naturalis regni vegetabilis. Vol. 4 Paris: Treüttel and Würtz; p. 55–220.

[CIT0004] Downie SR, Spalik K, Katzdownie DS, Reduron JP 2010 Major clades within *Apiaceae* subfamily *Apioideae* as inferred by phylogenetic analysis of nrDNA ITS sequences. Plant Divers Evol. 128:111–136.

[CIT0005] Kearse M, Moir R, Wilson A, Stones-Havas S, Cheung M, Sturrock S, Buxton S, Cooper A, Markowitz S, Duran C, et al. 2012 Geneious basic: an integrated and extendable desktop software platform for the organization and analysis of sequence data. Bioinformatics. 28:1647–1649. 22543367 10.1093/bioinformatics/bts199 PMC3371832

[CIT0006] Liu M, Downie SR 2017 The phylogenetic significance of fruit anatomical and micromorphological structures in Chinese *Heracleum* species and related taxa (*Apiaceae*). Syst Bot. 42:313–325.

[CIT0007] Logacheva MD, Valiejoroman CM, Degtjareva GV, Stratton JM, Downie SR, Samigullin TH, Pimenov MG 2010 A comparison of nrDNA ITS and ETS loci for phylogenetic inference in the Umbelliferae: an example from tribe Tordylieae. Mol Phylogenet Evol. 57:471–476. 20538066 10.1016/j.ympev.2010.06.001

[CIT0008] Mandenova IP 1959 Materialy po sistematike triby Pastinaceae K.-Pol. emend. Manden.(Umbelliferae –*Apioideae*) (Materials to systematics of tribe of Pastinaceae K.-Pol. bracts and bracteoles). Trudy Tbilissk Bot Inst. 20:3–57.

[CIT0009] Paik JH 2008 Systematic studies of *Heracleum* L. (Umbelliferae) and related genera in the Sino-Himalayan region [doctor thesis]. Edinburgh, UK: University of Edinburgh.

[CIT0010] Pu FD, Watson MF 2005 *Heracleum* L., *Semenovia* regel & herder and *Tordyliopsis* de candolle. *Apiaceae* In: Wu ZY, Raven PH, editors. Flora of China. Vol. 14 Saint Louis, China: Science Press; Beijing: Missouri Botanical Garden Press; p. 201.

[CIT0011] Stamatakis A 2014 RAxML version 8: a tool for phylogenetic analysis and post-analysis of large phylogenies. Bioinformatics. 30:1312–1313. 24451623 10.1093/bioinformatics/btu033 PMC3998144

[CIT0012] Xiao QY, Feng T, Yu Y, Luo Q, He XJ 2019 The complete chloroplast genome of *Semenovia gyirongensis* (Tribe Tordylieae, *Apiaceae*). Mitochondr DNA B. 4:1863–1864.

[CIT0013] Yu Y, Downie SR, He XJ, Deng XL, Yan L 2011 Phylogeny and biogeography of Chinese *Heracleum* (*Apiaceae* tribe Tordylieae) with comments on their fruit morphology. Plant Syst Evol. 296:179–203.

